# To the Editors of the Medical and Physical Journal

**Published:** 1808-12

**Authors:** 


*Impartiality, and a wish that every well-ascertained fact should be recorded, induce us to insert the following short communications as we received them.*

---

To the Editors of the Medical and Physical Journal.

Gentlemen,

Taking up, a few days ago, your Journal for September last, I observed a paper of Dr. Walker's, in which he supposes, (if I clearly understand him,) that the hydrophobic virus may lie dormant for a vast length of time in the part where it is inserted, and, finally, being disengaged from its "encystment," may come in contact with a living fibre, and then act on the system. This hypothesis has much plausibility in it, and I am induced to think it as satisfactory as any that can be offered. But, Sir, allow me to mention, that the same explanation was suggested years ago by Dr. Jenner among his disciples, with regard to the long-dormant state in which the vaccine virus remains after its insertion.

